# Potential Therapeutic Strategies for Renal Fibrosis: Cordyceps and Related Products

**DOI:** 10.3389/fphar.2022.932172

**Published:** 2022-07-08

**Authors:** Wei Tan, Yunyan Wang, Hongmei Dai, Junhui Deng, Zhifen Wu, Lirong Lin, Jurong Yang

**Affiliations:** ^1^ Nephrology, The Third Affiliated Hospital of Chongqing Medical University, Chongqing, China; ^2^ Nephrology, YunYang County People’s Hospital, Chongqing, China

**Keywords:** cordyceps, renal fibrosis, Chinese herbal medicine, chronic kidney disease, mechanism

## Abstract

At present, there is no effective drug for the treatment of renal fibrosis; in particular, a safe and effective treatment for renal fibrosis should be established. Cordyceps has several medical effects, including immunoregulatory, antitumor, anti-inflammatory, and antioxidant effects, and may prevent kidney, liver, and heart diseases. Cordyceps has also been reported to be effective in the treatment of renal fibrosis. In this paper, we review the potential mechanisms of Cordyceps against renal fibrosis, focusing on the effects of Cordyceps on inflammation, oxidative stress, apoptosis, regulation of autophagy, reduction of extracellular matrix deposition, and fibroblast activation. We also discuss relevant published clinical trials and meta-analyses. Available clinical studies support the possibility that Cordyceps and related products provide benefits to patients with chronic kidney diseases as adjuvants to conventional drugs. However, the existing clinical studies are limited by low quality and significant heterogeneity. The use of Cordyceps and related products may be a potential strategy for the treatment of renal fibrosis. Randomized controlled trial studies with good methodological quality, favorable experimental design, and large sample size are needed to evaluate the efficacy and safety of Cordyceps.

## Introduction

Renal fibrosis (RF) is a common outcome of the progression of various chronic kidney diseases (CKD) and the main pathological change in the progression of CKD to end-stage renal disease (Humphreys, 2018). It includes glomerulosclerosis (GS), renal interstitial fibrosis (RIF), and arteriosclerosis and perivascular fibrosis (Djudjaj and Boor, 2019). It shows histopathological features of excessive extracellular matrix (ECM) deposition, tubular atrophy, inflammatory cell infiltration, and loss of peritubular microvasculature. Subsequent structural destruction and functional impairment of the organ occurs, with scarring and sclerosis of the renal parenchyma. Cells involved in this event include renal tubular epithelial cells (TECs), endothelial cells (ECs), fibroblasts, pericytes, macrophages, and mast cells (Sun et al., 2016; Yan et al., 2021). In addition, cellular and molecular events such as inflammatory injury, oxidative stress, apoptosis, fibroblast activation, and epithelial–mesenchymal transition (EMT) are closely associated with RF (Liu et al., 2017; Nogueira et al., 2017). Despite significant progress in preclinical research of the mechanisms of RF and its therapeutic targets (Nogueira et al., 2017; Bai et al., 2021; Yan et al., 2021; Ruiz-Ortega et al., 2022), good target effects have not been demonstrated in clinical practice (Vincenti et al., 2017; Voelker et al., 2017). Therefore, there is still a lack of effective treatment that specifically targets RF. It is especially important to find safe and effective treatments for RF.

Chinese herbal medicines (CHMs) have been used for thousands of years in the treatment of kidney disease. Chinese herbs and fungi with potential kidney benefits include Cordyceps, Sairei-to, *Rheum* spp., *Salvia miltiorrhiza* and its components, and Magnesium lithospermate B (Wojcikowski et al., 2004). Anti-RF mechanisms of CHMs include anti-inflammatory and antioxidant effects, inhibition of EMT, reduction of ECM deposition, immune regulation, regulation of autophagy, inhibition of apoptosis, and control of hemorheology (Zhao et al., 2020). In this paper, we review the mechanisms of Cordyceps and related products in the treatment of RF (Table 1), as well as relevant clinical trials and meta-analyses (Table 2). We also discuss the possible nephrotoxicity of Cordyceps and the possible antifibrotic effects of the compounded formulation.

**TABLE 1:** Mechanisms of Cordyceps and related products against renal fibrosis.

Compound/extract	Animal/cell	Model/inducer	Outcomes	Targets/pathway	Mode of action	References
CCP	SD rats	HFD + STZ	TNF-α↓, IL-1β↓, IL-6↓	Blocking the TLR4/NF-κB and TGF-β1/Smad signaling pathway	Anti-inflammatory; regulation of dysbiosis of gut microbiota	Yang et al. (2020)
			Inflammatory cell infiltration↓			
			CTGF↓, MMP-2↓, HYP↓			
			Collagen deposition↓			
			a-SMA and Collagen I expression↓			
			Regulation of dysbiosis of gut microbiota			
	NRK-52E Cells	LPS	TNF-α↓, IL-1β↓, IL-6↓			
	NRK-49F cells	TGF-β	Collagen I↓, Fibronectin↓, α-SMA↓			
HEA	C57BL6/J mice	UUO	Integrity of renal parenchymal cells↑	Inhibition of TGF-β1/Smad and NF-κB signaling pathways	Anti-inflammatory	Zheng et al. (2018)
			Collagen deposition↓			
			TGF-β1↓, α-SMA↓, collagen I↓, Fibronectin↓			
			TNF-α↓, IL-6↓, IL-1β↓			
			M1↓, M2↑			
	RAW 264.7 cells	LPS	TNF-α↓,IL-1β↓, IL-10↑			
	NRK-49F cells	TGF-β	Fibronectin↓,α-SMA↓, collagen I			
HEA	SD rats	Alloxan monohydrate	TNF-α↓, IL-1β↓, IL-6↓	Inhibition of TGF-β1 and NF-κB	Anti-inflammatory; antioxidative stress	Wang et al. (2019)
			SOD↑, CAT↑, GSH↑, MDA ↓			
			Tubular necrosis↓, inflammatory infiltration↓, thickening of the membrane basement↓			
Cordycepin	HEK293T cells	—	IL-1 β↓, TNF-α↓	Inhibition of TLR4/NF-κB pathway	Anti-inflammatory	Sun et al. (2019)
Cordyceps cicadae/Paecilomyces cicadae	SD rats	250 mg/kg adenine	MDA↓, GSH↓, SOD↑	May be related to the downregulated PAI-1	Antioxidative stress; anti-inflammatory	Li et al. (2019)
			Kidney indices↓, the number of the glomerulus↑			
			Interstitial inflammation in the proximal and distal convoluted tubules↓			
Ophiocordyceps Lanpingensis	C57BL/6 Mice	0.25% adenine	Ameliorate the CKD pathological Changes	Inhibition of TLR4-mediated MAPK/NF-κB pathway	Antioxidative stress; anti-inflammatory Antiapoptosis	Zhou et al. (2021)
			Recruitment of macrophages↓			
			SOD↑, GSH-PX↑, ROS↓, MDA↓			
			TNF-α↓, IL-1β↓, IL-10↑			
			Apoptosis cells↓, BAX↓, Caspase-3↓, Caspase-9↓, Bcl-2↑			
			Tissue fibrosis↓, TGF-β1 expression↓, α-SMA↓, collagen I↓			
HEA	HK-2 cells	NSAIDs diclofenac/meloxicam	ROS↓	Regulates the GRP78/ATF6/PERK/IRE1α/CHOP pathway	Antioxidative stress; antiapoptosis	Chyau et al. (2021)
			Apoptosis cells↓			
*Cordyceps militaris*	SD rats	HFD + STZ	SOD↑, GSH-px↑, ROS↓, MDA↓	Phosphor-AKT↓ phosphor-GSK-3β↓	Antioxidative stress; anti-inflammatory	Liu et al. (2016)
			Inflammatory infiltrate↓			
*Cordyceps cicadae*	SHR/WK rats	SHR	Collagen deposition↓, a-SMA↓	Inhibition of SIRT1/p53 pathway	Antiapoptosis	Huang et al. (2020)
	Primary human RPTEC	AngII	Apoptosis cells↓, Caspase-3↓			
CMP	C57BL/6 mice	STZ	Collagen deposition↓	—	Activation of autophagy; anti-inflammatory; antioxidative stress	Chen et al. (2019a)
			CD68↓, IL-1β↓, IL-6↓, MCP-1↓			
			Autophagosome↑			
			Atg5↑, beclin1↑, LC3↑, p62↓			
			GSH↑, MDA↓			
*Cordyceps cicadae*	SHR/WK rats	SHR	Collagen deposition↓	Activation of SIRT1/FOXO3a/ROS	Inhibition of autophagy; antioxidative stress	Cai et al. (2021)
			TGFβ1↓, α-SMA↓, fibronectin↓, collagen I↓			
			Apoptosis cells↓			
			LC3II↓, beclin-1↓, SQSTM1/p62↓			
			SOD↑, MDA ↓			
	NRK-52E cells	AngII/rapamycin	Collagen I↓, α-SMA↓			
			LC3II↓, beclin-1↓			
Nucleoside/nucleobase extract	C57BL/6 mice	STZ	Collagen deposition↓	Phosphor-p38↓ phosphor-ERK↓	Inhibition of EMT and ECM deposition	Dong et al. (2019)
			Fibronectin↓, collagen I↓			
			E-cadherin↑, α-SMA↓			
	HK-2 cells	HG	Morphological and phenotypic changes↓			
			E-cadherin↑, α-SMA↓			
			Fibronectin↓, collagen I↓			
Ergosterol peroxide	NRK-49F cells	TGF-β1	Renal fibroblast proliferation rate↓	Blocking the phosphorylation of ERK1/2, p38, and JNK pathway	Ameliorates TGF-β1-induced activation of kidney fibroblasts	Zhu et al. (2014)
			Fibronectin↓, a-SMA↓, vimentin↓, CTGF↓			
CmNo1 extract	C57BL/6J mice	HFD + STZ	Collagen IV↓	TGF-β1↓	Inhibition of collagen deposition	Yu et al. (2016)
*Cordyceps sinensis*	SD rats	5/6 nephrectomy	Interstitial fibrosis↓	Inhibition of TGF-β1/Smad pathway	Inhibition of EMT and ECM deposition	Pan et al. (2013)
			E-cadherin↑, α-SMA↓, FSP1↓			
			TGF-β1↓, TβR1↓, TβR2↓, Smad2↓, Smad3↓, Smad7↑			
3′-Deoxyadenosine	C57BL/6 mice	UUO	Collagen I↓, α-SMA↓	Reduced phosphorylation and total Smads through transcriptional repression	Inhibition of ECM deposition	Gu et al. (2013)
			Interstitial myofibroblasts↓, fibrotic area↓			
	NRK-52E	TGF-β/BMP-4	Collagen I↓, collagen IV↓			
*Cordyceps sinensis*	SD rats	UUO	Interstitial collagen deposition Masson↓, α-SMA↓	BAG3↓	Inhibition of EMT and ECM deposition	Du et al. (2015)
*Hirsutella sinensis*	C57BL/Ks mice	db/db diabetic	Interstitial fibrosis↓	Metabolites of the TCA cycle, glycolysis, pentose phosphate pathway, pyrimidine metabolism, and purine metabolism↓	Regulation of disturbed metabolome	Lu et al. (2019)
			TGF- β1↓, CTGF↓, VEGF↓, fibronectin↓, collagen I↓, collagen IV↓			

HFD, high-fat diet; TNF, tumor necrosis factor; IL-1, interleukin 1; IL-6, interleukin 1; CTGF, connective tissue growth factor; MMP-2, matrix metalloproteinase-2; HYP, hydroxyproline; a-Smooth muscle actin, a-shape memory alloy; TLR4, Toll-like receptor 4; NF-κB, Nuclear factor-kappaB; TGF-β1, transforming growth factor beta1; SOD, superoxide dismutase; CAT, catalase; GSH, glutathione; MDA, malondialdehyde; ROS, reactive oxygen species; Bcl-2, B-cell chronic lymphocytic leukemia/lymphoma-2; BAX, Bcl-2-associated X protein; GRP78, glucose-regulated protein 78; ATF6, activating transcription factor 6; PERK, protein kinase R (PKR)-like endoplasmic reticulum kinase; IRE1α, inositol-requiring enzyme 1 alpha; CHOP, C/EBP homologous protein; SHR, spontaneously hypertensive; RPTEC, renal proximal tubular epithelial cells; WK, wistar-kyoto; SIRT1, Sirtuin-1; MCP-1, monocyte chemoattractant protein-1; Atg5, Autophagy Protein 5; LC3, light chain 3; SQSTM1, sequestosome1; FOXO3a, forkhead box class O3a; HG, high glucose; ERK, extracellular signal-regulated kinase; JNK, c-Jun N-terminal kinase; CmNo1, cordyceps militaris No.1; FSP1, ferroptosis suppressor protein 1; EMT, epithelial–mesenchymal transition; ECM, extracellular matrix; BAG3, Bcl-2-associated athanogene 3; VEGF, vascular endothelial growth factor; TCA, tricarboxylic acid.

**TABLE 2:** Clinical studies with Cordyceps.

Study type	N	Therapeutic arms	Disease	Duration	Outcomes	References
RCT	98	COG vs. CG	CKD late stage 3 or stage 4	3 months	In COG group	Sun et al. (2019)
					Inflammatory state and thickness of glomerular filtration membrane in renal tissue↑	
					Urinal protein↓ BUN↓, creatinine↓, EGFR↑ (*p* < 0.05)	
					TG↓, TC↓, LDL-C↓, HDL-C↑ (*p* < 0.05)	
					Cys-C ↓, MPO ↓, MDA ↓, NO ↑, SOD ↑ (*p* < 0.05)	
Study cohort	160	Bailing capsules + losartan vs. losartan	Diabetic glomerulosclerosis	—	In Bailing capsules + losartan group	Yu et al. (2021)
					ORR↑, 91.25% vs. 78.75%	
					DBP↓, SBP↓, Scr↓, BUN↓, 24 h UP↓, mALB↓, β2-MG↓,GFR↑ (*p* < 0.01)	
					TCM points↓ (*p* < 0.01)	
					SOD↑, ROS↓, 8-OHdG↓, hs-CRP↓, TGF-β1↓, SAA↓ (*p* < 0.01)	
Review	1746	Cordyceps vs. placebo, no treatment, or conventional treatment	CKD	—	In Cordyceps group	Zhang et al. (2014)
					Scr↓ (14 studies, 987 participants), Ccr↑ (6 studies, 362 participants), 24 h UP ↓ (4 studies, 211 participants)	
Meta-analysis	3955	JSB capsules + ACEI/ARB vs. ACEI/ARB	Diabetic kidney disease	28 days–4 weeks	In JSB combined with ACEI/ARB group	Li and Xu, (2020)
					ORR↑ (OR 4.91; 95% CI 3.32–7.25)	
					24 h UP↓, UAER↓, Scr↓, BUN↓ (*p* < 0.0001)	
					SBP↓, DBP↓,FBG↓, HA1c↓, TC↓, TG↓	
Meta-analysis	2198	JSB + ARBs vs. ARBs	Early diabetic Nephropathy	8–28 weeks	In JSB + ARBs group	Lu et al. (2018)
					ORR ↑ (OR 3.84; 95% CI: 2.37–6.24; *p* < 0.000001)	
					24 h UTP↓, UAER↓, ACR↓, Cys-C↓, TG↓ (*p* < 0.000001),	
					BUN↓ (*p* = 0.005), Scr↓ (*p* = 0.0006), SBP↓ (*p* = 0.0001), DBP↓ (*p* = 0.03)	
Meta-analysis	4288	*Ophiocordyceps sinensis* + ACEI/ARB vs. ACEI/ARB	Diabetic kidney disease	4–24 weeks	In *Ophiocordyceps sinensis* + ACEI/ARB group	Luo et al. (2015)
					24 h UP↓, UAER↓, MAU↓, CRP↓, TG↓, TC↓ (*p* < 0.000001)	
					BUN↓, Scr↓ (*p* < 0.0001)	
					SBP↓ (*p* = 0.006)	
Meta-analysis	1941	Bailing capsules + control group treatment vs. routine treatment, and/or combined with Western medicine	Type 2 diabetic nephropathy	4–24 weeks	In the group containing Bailing capsules	Sheng et al. (2020)
					ORR ↑ (OR = 1.24; 95% CI: 1.11–1.38)	
					24 h urine total protein↓, UAER↓, Scr↓, BUN↓ (*p* < 0.01)	
RCT	97	HEA-enriched *Cordyceps cicadae* Mycelium vs. placebo	—	3 months	No differences in symptoms or side effects between participants. There was also no significant difference between baseline measurements and the final analysis of biochemical analysis, such as kidney function, liver function, and blood electrolytes	Tsai et al. (2021)

COG, cordycepin group; CG, control group; GFR, glomerular filtration rates; TG, triglyceride; TC, total cholesterol; LDL-C, low-density lipoprotein cholesterol; HDL-C, low-density lipoprotein cholesterol; Cys-C, cystatin-C; MPO, myeloperoxidase; MDA, malondialdehyde; NO, nitric oxide; SOD, superoxide dismutase; DBP, diastolic blood pressure; SBP, systolic blood pressure; Scr, serum creatinine; BUN, blood urea nitrogen; 24 h UP, 24 h urine protein; mALB, urine microalbumin; β2-MG, β2 microglobulin; TCM, traditional chinese medicine; ROS, reactive oxygen species; 8-OhdG, 8-hydroxydeoxyguanine; hs-CRP, hypersensitive C-reactive protein; TGF-β1, transforming growth factor β1; SAA, serum amyloid A; Ccr, creatinine clearance; FBG, fasting blood glucose; HA1c, hemoglobin A1c; JSB, jinshuibao; CI, confidence interval; MD, mean difference; ORR, overall response rate; ACEI, angiotensin-converting enzyme inhibitors; ARB, angiotensin receptor blockers; 24 h UTP, 24 h urinary total protein; UAER, urinary albumin excretion rate; ACR, albumin-to-creatinine ratio; MAU, microalbuminuria.

Cordyceps is a fungus that colonizes the larvae of moths. It has been used for centuries as a medicine in China, Japan, and other Asian countries. It has several species, including *Cordyceps sinensis*, *Cordyceps militaris*, and *Cordyceps cicadae*. Cordyceps has a variety of medicinal properties or bioactive compounds, including nucleosides, polysaccharides, cyclodepsipeptides, sterols, alkaloids, and phenolics (Olatunji et al., 2018), which show immunomodulatory, antitumor, anti-inflammatory, antioxidant, renoprotective, and other effects (Das et al., 2020). Cordyceps has shown potential promise as an adjunct to conventional medicine to decrease serum creatinine (Scr), increase creatinine clearance, reduce proteinuria, and alleviate CKD-related complications (Zhang et al., 2014). Based on the network pharmacology tools that are used to investigate the molecular mechanism of Cordyceps for the treatment of diabetic nephropathy (DN), seven active ingredients were screened from Cordyceps, 293 putative target genes were identified, and 85 overlapping targets matching DN were identified as potential therapeutic targets, such as tumor necrosis factor (TNF), mitogen-activated protein kinase 1, epidermal growth factor receptor (EGFR), angiotensin-converting enzyme, and Caspase-9. Cordyceps are involved in an inflammatory response, apoptosis, oxidative stress, insulin resistance, and other biological processes through these pathways (Li et al., 2021).

## Mechanisms of Cordyceps and Related Products Against Renal Fibrosis

### Cordyceps Relieves Inflammation

Inflammation is the initiator of RF, and persistent chronic inflammation is considered a hallmark feature of CKD (Meng, 2019). In response to pathogenic factors, damaged renal TECs recruit inflammatory cells to the renal interstitial region, and these inflammatory cells then produce large amounts of proinflammatory and profibrotic cytokines (Gewin et al., 2017). Various active ingredients in Cordyceps have anti-inflammatory effects (Phull et al., 2022). They have been shown to protect against liver fibrosis through anti-inflammatory effects (Xu et al., 2021) (Ying-Mei et al., 2020). Cordyceps extract attenuates renal histological changes, reduces Scr and blood urea nitrogen (BUN) levels, and decreases inflammatory factors in acute kidney injury (AKI), including renal ischemia/reperfusion injury (Han et al., 2020) and cisplatin-induced AKI (Deng et al., 2020). In RF related to streptozotocin (STZ), or alloxan monohydrate–induced DN, the active components of Cordyceps, *Cordyceps cicadae* polysaccharides (CCP) or N^6^-(2-hydroxyethyl) adenosine (HEA), are effective in reducing the expression of proinflammatory factors TNF-α, IL-1β, and IL-6 in serum and kidney and in reducing the expression of interstitial fibrosis-associated proteins α-SMA and collagen I (Wang et al., 2019; Yang et al., 2020). *In vitro*, CCP also reduces the release of lipopolysaccharide-induced proinflammatory factors and TGF-β-induced activation of fibroblasts (Yang et al., 2020). Unilateral ureteral obstruction (UUO) is a classical RF model, and Cordyceps extract HEA reduces fibrosis-associated proteins TGF-β1, α-SMA, collagen I, and fibronectin, and inflammatory factors TNF-α, IL-6, and IL-1β in renal tissue 14 days after UUO (Zheng et al., 2018). Zheng et al.’s (2018) study initially explored the effect of HEA on inflammatory cells and found that HEA blocked the accumulation of M1 macrophages and induced the accumulation of M2 macrophages in the kidney as reflected by the positive detection of the F4/80 antigen. The role of M2 macrophages in fibrosis is controversial (Ricardo et al., 2008) (Lin et al., 2010), but the M1/M2 macrophage ratio is an important stage in the development of RIF. However, this study did not further distinguish between the subtypes of M2.

The abovementioned studies have explored the regulatory mechanism of Cordyceps extract against inflammation in RF by inhibiting the TGF-β1/Smad and TLR4/NF-κB signaling pathways (Zheng et al., 2018; Yang et al., 2020). Sun et al. found that p-TLR4, TLR4, p-NF-κB, NF-κB, IL-1 β, and TNF-α levels were effectively reduced by Cordycepin treatment in human embryonic kidney 293T cells. However, Cordycepin did not reduce the levels of these molecules when TLR4 was silenced. These results suggest that Cordycepin may affect the NF-κB signaling pathway through TLR4 (Sun et al., 2019). TLRs/NF-κB activate the downstream inflammasome NOD-like receptor family pyrin domain containing 3 (NLRP3) (Xue et al., 2019), which promotes the maturation of proinflammatory factors IL-1β and IL-18 by activating Caspase-1 (Liston and Masters, 2017). Multiple studies have shown that the NLRP3 inflammasome and its downstream pyroptosis and inflammation play an important role in the development of RF (Ma et al., 2022) (Ram et al., 2022; Wang et al., 2022). It has been shown that *Ophiocordyceps sinensis* causes inhibition of mRNA and protein expression of NLRP3 inflammasome and downstream effectors IL-1β and IL-18 in a rat model of DN (Wang et al., 2018). Thus, Cordyceps and related products are effective in relieving inflammation in RF through multiple mechanisms.

### Cordyceps Attenuates Oxidative Stress

The kidney is a highly metabolically active organ with mitochondria rich in oxidative reactions and susceptible to oxidative stress damage. Oxidative stress and inflammation interact to play a key role in renal tissue destruction, irreversible loss of renal function, and progression of RF (Xu et al., 2015; Darby and Hewitson, 2016; Richter and Kietzmann, 2016). Normal cells produce small amounts of reactive oxygen species (ROS), which play an important physiological role. Free radical–scavenging enzymes and antioxidants maintain oxygen metabolism homeostasis by activating transcription factors, regulating physiologically active substances and inflammatory immunity, and promoting cell proliferation and differentiation. Oxidative stress occurs when the balance between ROS and reactive nitrogen species and the antioxidant defense system is disrupted, that is, when the production of pro-oxidants or ROS exceeds the endogenous antioxidant capacity (Sies et al., 2017). It has been shown that oxidative stress promotes the progression of RF (Aranda-Rivera et al., 2021); if oxidative stress is suppressed, RF can be attenuated (Liao et al., 2022; Lo et al., 2022). Antioxidant enzymes, such as superoxide dismutase (SOD), catalase, and glutathione peroxidase (GSH-Px), protect cells from damage caused by oxygen free radicals. Malondialdehyde (MDA) is one of the products of the reaction between lipids and oxygen free radicals, and it accumulates during oxidative stress. Cordyceps improves the redox properties of CKD by affecting the levels of NO, SOD, and MDA in serum. In rat models of DN (Liu et al., 2016; Wang et al., 2019) and membranous nephropathy (Song et al., 2016), both *C. militaris* and HEA showed excellent ability to attenuate oxidative stress, elevated SOD and GSH, and decreased MDA levels. Using diclofenac or meloxicam to induce oxidative stress, HEA intervention significantly reduced the level of ROS in human proximal tubular cells (HK-2) cells (Chyau et al., 2021). In rats with adenine-induced chronic renal failure, *Cordyceps cicadae* and *Paecilomyces cicadae* effectively reduced serum urea and creatinine levels, improved renal histopathology, inhibited oxidative stress, and enhanced antioxidant capacity (Li et al., 2019). In a CKD mouse model established by adenine gavage, *Ophiocordyceps lanpingensis* polysaccharides elevated SOD and GSH-PX, decreased ROS and MDA, improved histopathological staining, and decreased fibrosis-related proteins TGF-β1, α-SMA, and collagen I (Zhou et al., 2021). Cordyceps and related products have a favorable ability to attenuate oxidative stress, but the mechanisms involved are not well understood.

### Cordyceps Inhibits Apoptosis

A variety of kidney injury factors may trigger apoptosis, including ischemia/reperfusion injury (Xu et al., 2019), cisplatin-induced kidney injury (Yang et al., 2018), and DN (Peng et al., 2015). Apoptosis (a programmed cell death) of glomerular ECs, podocytes, and TECs is closely associated with RF (Thomas et al., 1998; Docherty et al., 2006). Inhibition of apoptosis as a therapeutic target can alleviate fibrosis (Chen et al., 2021; Xia et al., 2021; Park et al., 2022). Cordyceps downregulates apoptosis in ischemia/reperfusion injury and Cyclosporine A–induced renal tubular dysfunction (Shahed et al., 2001; Chyau et al., 2014). Apoptosis is also considered an important mechanism in contrast-induced nephropathy (CIN). *C. sinensis* prevents CIN in diabetic rats by decreasing the expression of apoptosis-related proteins Caspase-3 and Bax and increasing the expression of antiapoptotic protein Bcl-2. Mechanistically, *C. sinensis* decreases the expression of JNK protein and increases the expression of ERK protein (Zhao et al., 2018). Renal tubular interstitial fibrosis is a typical pathological feature of hypertensive kidney injury. In a spontaneous hypertension rat model, *C. cicadae* reduces interstitial fiber deposition, α-SMA expression, apoptosis, and Caspase-3 activity by regulating the SIRT1/p53 signaling pathway (Huang et al., 2020). *In vitro*, the major renal damage caused by hepatitis B virus infection is hepatitis B virus X (HBx)–induced apoptosis of renal TECs, which is related to the increased Caspase-3 and Caspase-9 activity and increased PI3K/Akt pathway activity. *C. sinensis* attenuates all of these HBx-induced responses, at least in part by inhibiting the PI3K/Akt signaling pathway (He et al., 2020). Cordyceps and related products have been shown to inhibit apoptosis *in vivo* and *in vitro*, but the mechanisms involved have not been unified.

### Cordyceps Regulates Autophagy

Autophagy is a “self-consuming” cell death pathway that degrades most cytoplasmic components by forming autophagosomes and autolysosomes (Yang and Klionsky, 2010). Basal autophagy in the kidney is critical for maintaining renal homeostasis, structure, and function. Namely, basal autophagy removes potentially dysfunctional organelles and long-lived proteins to maintain cellular homeostasis. In response to environmental and intracellular stress, autophagy may serve as an adaptive response to ensure cell survival. However, autophagy may also play a role in cellular dysfunction and organ lesions (Tang et al., 2020), such as AKI (Kaushal and Shah, 2016), AKI-CKD (Baisantry et al., 2016), and DN (Lenoir et al., 2015). The role of autophagy in RF is controversial. It has been reported that sustained activation of autophagy in renal tubular cells promotes interstitial fibrosis, and inhibition of autophagy with chloroquine or selective deletion of Atg7 in proximal tubules alleviates RF in UUO mice (Livingston et al., 2016). Several studies have also reported antifibrotic effects of autophagy, whereas inhibition of autophagy by 3-methyladenine exacerbated RF in a UUO rat model, suggesting that autophagy may exert antifibrotic effects by attenuating renal tubular cell injury (Kim et al., 2012). In STZ-induced DN mice, *C. militaris* polysaccharides (CMP) decrease collagen deposition in the kidney and increase the rate of autophagy; promote the expression of autophagy-specific protein Atg5, Beclin1, and LC3; and decrease the expression of p62 protein in the kidney. This suggests that CMP administration significantly enhances autophagy (Chen et al., 2019a). In a hypertensive nephropathy model, *Cordyceps cicadae* may attenuate the expression of fibrosis-associated proteins. It ameliorates RF induced by hypertension by downregulating autophagy-related proteins LC3II and Beclin-1, thereby significantly inhibiting autophagic vesicles and attenuating autophagic stress. This effect may be attributed to the regulation of SIRT1 pathway-mediated autophagic stress and is achieved by modulating the autophagy regulator forkhead box class O3a (FOXO3a) and oxidative stress (Cai et al., 2021). The regulatory effect of Cordyceps on autophagy is indisputable, but autophagy itself is diverse and can be divided into microautophagy, chaperone-mediated autophagy, and macroautophagy, and macroautophagy can be divided into nonselective autophagy and selective autophagy depending on whether the substrate is selective or not. Because autophagy is environment dependent and may play different roles at different stages of the disease, different cell types do not respond consistently to the autophagy activation, resulting in a bidirectional effect of cellular autophagy on the process of RF. The regulatory role of autophagy in fibrosis remains to be further investigated.

### Cordyceps Reduces Extracellular Matrix Deposition and Fibroblast Activation

Aberrant activation and proliferation of fibroblasts are believed to be key causes of the progression of RF (Zhou et al., 2017). Activated fibroblasts, which promote the production and release of ECM collagens I, III, and IV and fibronectin (Sun et al., 2016), are the main source of ECM during scar tissue formation. Myofibroblasts in RF can be derived from mesenchymal fibroblasts, bone marrow–derived fibroblasts, renal TECs, ECs, pericytes, and macrophages. TECs can produce stroma through the EMT to fibroblasts and myofibroblasts (Yan et al., 2021). In this process, TGF-β is the main driving factor. TGF-β induces fibroblast activation and proliferation as well as excessive synthesis and accumulation of ECM through downstream signaling pathways such as SMAD, BMMP-7, and CTGF, thereby promoting fibrosis. Targeted inhibition of TGF-β and its downstream signaling pathways delay fibrosis (Meng et al., 2016; Walton et al., 2017; Ma and Meng, 2019). In STZ-induced diabetic mice and high glucose–exposed HK-2 cells, the nucleoside/nucleobase–rich extract from Cordyceps increased E-cadherin expression; decreased α-SMA, fibronectin, and collagen I expression; inhibited EMT and ECM deposition; and improved the fibrotic morphology of tissues and cells. Cordyceps extract effectively inhibited the phosphorylation of p38 and ERK, did not affect the phosphorylation level of JNK, and had a synergistic effect on fibrosis with p38 or ERK inhibitors (Dong et al., 2019). Ergosterol peroxide from *Cordyceps cicadae* improves TGF-β1-induced renal fibroblast activation *via* the mitogen-activated protein kinases signaling pathway (Zhu et al., 2014). In DN C57BL/6J mice and 5/6 nephrectomy rats, *C. militaris* (Yu et al., 2016) and *C. sinensis* (Pan et al., 2013) counteracted RF and reduced the expression of fibrosis-associated proteins by inhibiting the TGF-β1 pathway. Further exploring downstream mechanisms, 3′-deoxyadenosine interfered with TGF-β and bone morphogenetic protein signaling by downregulating Smads at the transcriptional level. In that way, it induced decreases in collagen I and α-SMA, mesenchymal myofibroblast number, and fibrotic area (Gu et al., 2013). Cordyceps and its extracts are potential therapeutic strategies against fibrosis *via* the TGF-β/Smad and other pathways.

### Other Mechanisms

In addition to the above mechanisms, *C. sinensis* attenuates RF in the UUO model by inhibiting Bcl-2-associated athanogene 3 (BAG3), which has been reported to be involved in cell proliferation, apoptosis, adhesion and migration, and EMT processes (Du et al., 2015).

Recent studies have reported that gut microbiota mediates RF (Liu et al., 2021). CMP reduce renal injury by regulating intestinal flora imbalance (Song and Zhu, 2020). In a study of CCP to attenuate fibrosis in DN, high-throughput pyrophosphate sequencing of 16S rRNA indicated that CCP regulated dysbiosis of the intestinal flora by increasing the relative abundance and proliferation of probiotic bacteria, thereby exerting a beneficial effect on tubulointerstitial fibrosis in DN rats (Yang et al., 2020).

Normal energy metabolism is particularly important for maintaining the structure and function of the kidney, and metabolic reprogramming is generally considered a key process in activating fibrosis in different organs, as it is in the kidney (Barcena-Varela et al., 2021). The balance of fatty acid oxidation and glycolysis directly affects ECM production and degradation or indirectly affects RF through inflammation and hypoxia (Zhu et al., 2021). Metabolomic analysis has been used to explore the metabolic regulatory effects of *C. sinensis* in a db/db diabetic mice model. *Hirsutella sinensis*—the anamorph of the traditional Chinese medicine *C. sinensis*—attenuates RF, ameliorates system-wide disorders of glucolipid metabolism and amino acid deficiency, and regulates excessive activation energy and nucleotide metabolism (Lu et al., 2019). This suggests that Cordyceps may have a potential role in the regulation of renal metabolism and, consequently, may ameliorate fibrosis.

## Clinical Studies of Cordyceps and Related Products in Treating Chronic Kidney Diseases

In this section, we review published clinical studies of Cordyceps and related products, including two randomized controlled trial (RCT) studies, one cohort study, one review, and four meta-analyses (Table 2).

One study recruited 98 patients with CKD3 or CKD4 and randomized them to the Cordycepin group (COG, patients received 100 mg of *C. militaris* per day) and the control group (CG, patients received dried chickweed herb placebo per day). After 3 months of treatment, *C. militaris* improved the inflammatory status and thickness of the glomerular filtration membrane of renal tissues. Compared with the CG group, the COG group had lower CKD biomarkers (*p* < 0.05); higher EGFR (*p* < 0.05); lower serum Cys-C, MPO, and MDA levels; and higher NO and SOD levels (*p* < 0.05). *C. sinensis* reduced protein and mRNA levels of the TLR4/NF-κB signaling pathway (*p* < 0.05). This suggests that *C. militaris* improves CKD by affecting the TLR4/NF-κB redox signaling pathway (Sun et al., 2019).

To evaluate the efficacy of the Cordyceps preparation Bailing capsule combined with losartan in the treatment of diabetic GS, a cohort that included 160 patients with diabetic GS was randomly divided into the observation and the CGs; the observation group was treated with losartan and Bailing capsules, and the CG was treated with losartan. The overall effective rate was higher in the observation group than in the CG (*p* < 0.05). After the treatment, the DBP, SBP, Scr, 24 h UP, BUN, mALB, and β2-MG levels were lower, and the GFR was higher in the observation group than in the CG (*p* < 0.01). The traditional Chinese medicine symptom scores were lower in the observation group than in the CG (*p* < 0.01). The observation group also showed higher serum SOD and lower ROS, 8-OHdG, hs-CRP, TGF-β1, and SAA levels than the control group (*p* < 0.01). Bailing capsules combined with losartan improved the efficacy; improved the blood and urine biochemical indexes, the renal function, and the clinical symptoms; and reduced the oxidative stress. However, this study has the disadvantages of small sample size, short follow-up time, and bias in case selection (Yu et al., 2021).

In another review, 22 studies involving 1,746 participants were included. *C. sinensis* was found to significantly reduce Scr, increase creatinine clearance, and reduce 24 h proteinuria in patients with CKD. All 22 studies were published in Chinese and conducted in Chinese hospitals. The quality of evidence from the included trials in the evaluation was suboptimal; namely, four trials had a high risk of bias because of the lack of clear description of randomization, allocation concealment, and blinding, whereas the remaining 18 trials had an unclear risk of bias. In some of the included trials, there was significant heterogeneity in the conventional treatment as a co-intervention. Since there was no blinding during the study, possible differences in the multiple combined interventions between the treatment and control groups may have introduced bias into the results. Therefore, the poor methodological quality and underreporting of the included studies meant that no definitive conclusions could be drawn regarding the possible effects of Cordyceps preparations in patients with CKD (Zhang et al., 2014).

Four meta-analyses on Cordyceps preparations (JinShuiBao capsule/Bailing capsule) for DN included 51 RCT studies consisting of 3,955 participants (Li and Xu, 2020), 26 RCT studies with 2,198 early DN participants (Lu et al., 2018), 60 studies with 4,288 participants (Luo et al., 2015), and 24 studies with 1,941 participants (Sheng et al., 2020). All four studies showed that the Cordyceps preparation combined with ACEI/ARB was superior to ACEI/ARB alone, as shown by increased overall response rate and decreased 24 h UTP, UAER, BUN, and Scr. None of the studies had serious publication bias. However, all of the participating patients were Chinese with no other ethnicity; most of the included RCTs did not report detailed methodologies; in addition, efficacy assessments were not standardized and rigorous. The low quality of the study design and significant heterogeneity reduced the credibility of the meta-analysis.

To assess the safety of *C. cicadae*, 97 healthy adults were randomized to the treatment group (*n* = 49) and the placebo group (*n* = 48) in a double-blind, randomized trial. The treatment group received 1.05 g of *C. cicadae* mycelium granules once a day after a meal for 3 months. The placebo group received 1.05 g granules with the same nutrition, but without additional *C. cicadae* mycelium. Blood samples were collected before and after the treatment for biochemical analysis, including renal function, liver function, blood lipid, and electrolyte levels. No differences in symptoms or side effects were observed between the participants in the trial. There were also no significant differences between the baseline measurements and the final biochemical analyses. The results of the trial indicate that it is safe to consume less than 1.05 g of HEA-rich mycelium per day (Tsai et al., 2021).

In addition to single drugs or extracts, there are many compound preparations of Chinese herbs; some of the compound formulations of Cordyceps also have renal protective effects. WH30+ is a Chinese herb preparation composed of *Rheum palmatum*, *S. miltiorrhiza*, *C. sinensis*, *Leonurus sibiricus*, *Epihedium macranthum*, Radix Astragali, and Radix Codonopsis Pilosulae. It has nephroprotective effects in both glycerol-induced acute renal failure and adenine-induced chronic renal failure rats (Ngai et al., 2005). Cordyceps-related formulations of Novel JY5 formula (Fu et al., 2021), CGA formula (Li et al., 2016; Tian et al., 2019), and Fuzheng Huayu formula (Chen et al., 2019b) all have good effects against liver fibrosis, suggesting that these formulations may also be potential novel therapeutic candidates for RF.

## Conclusion and Perspective

In this study, we found that Cordyceps and related products could attenuate RF through multiple pathways and targets, but the mechanisms were not independent of each other. Targeting TLR4 could reduce inflammation and oxidative stress *via* NF-κB or activate the downstream TGF-β/Smad signaling pathway to promote ECM deposition and fibroblast activation. Acting on SIRT1 was important in the regulation of autophagy, oxidative stress, energy homeostasis, and apoptosis. Although multitargeting had certain advantages, it could also have more side effects. Toxic side effects associated with Cordyceps have been reported in animals and humans. *C. militaris* was administered to rats at doses of 0, 1, 2, and 3 g/kg per day. Nephrotoxicity characterized by renal tubular epithelial degeneration and necrosis occurred at high doses of 3 g/kg, and the male rats were more susceptible to nephrotoxicity than female rats (Zhou and Yao, 2013). Doan et al. reported 60 cases of apparent Cordyceps fungus poisoning occurring in southern Vietnam during the rainy seasons, between 2008 and 2015. All patients showed symptoms within 60 min of consuming cicada flowers. A 60-year-old male patient died after consuming five cicada flowers and ingesting approximately 200 ml of rice wine. Another patient ingested 15–16 cicada flowers; although he exhibited severe symptoms, he survived after 3 weeks of treatment. These observations suggest that there are individual differences in the doses of cicada flower poisoning (Doan et al., 2017). It is therefore crucial to establish more systematic, complete, and feasible pharmacological and toxicological research methods.

Based on the available preclinical studies, Cordyceps and related products are supported for the treatment of RF. However, the available clinical data are limited. The published study protocol has been improved (Hu et al., 2021); as such, future RCTs with good methodological quality, favorable experimental design, and large sample size are needed to explore the therapeutic effects of Cordyceps and related products on RF to provide more rigorous clinical study data.
